# Collateral circulation in the obstruction of the superior vena cava flow

**DOI:** 10.1080/07853890.2021.1897447

**Published:** 2021-09-28

**Authors:** J. Ovelar, J. Cédola, J. Merino

**Affiliations:** CIMED FUNDAMI Digital Anatomy Laboratory, La Plata, Buenos Aires, Argentina

## Abstract

**Introduction:**

By means of virtual reconstructions obtained from the data provided by the different Multislice Computed Venotomographies (MCVT) [1--3] performed in our institution during the period from 2015 to date, a total of 14 patients with different pathologies (oncological, venous thrombosis by port-a-cath catheter, etc.) were evaluated, which showed signs of obstruction of the superior vena cava and collateral circulation. The aim is demonstrating a venous shunt pattern in cases of Superior Vena Cava obstruction by virtual representation of the collateral circulation in acute or chronic stage.

**Materials and methods:**

Sixtty-four detector Phillips Multislice tomograph and process the data using IntelliSpace Portal (specialized Phillips software).

**Results:**

The collateral circulation network chosen depended mainly on whether or not the Azygous vein was compromised and the time of evolution of the obstruction [4]. From this, it is possible delimit two circuits: an anterior collateral drainage and a posterior collateral drainage. The posterior circuit is presented principally in acute cases, with the indemnity of the root of the Azygous Vein and through the intercostal veins, vertebral plexuses, the Azygos system, Hemi-azygous, and accessory. Whereas, the anterior circuit is mainly presented by the obstruction of the Superior Vena Cava and the Azygous too, or in chronic situations of obstruction of the SVC [5], either by means of anterior thoracic collateral, middle and/or lateral, that through superficial or deep venous tributaries return the venous flow to the central circulation by the Inferior Vena Cava. **Discussion and conclusions:** The knowledge of the venous anatomy, through virtual representations, allow to understand the collateral circulation and its patterns in cases of obstruction of the Superior Vena Cava [6].
